# Relative validation of a food frequency questionnaire for national health and nutrition monitoring

**DOI:** 10.1186/1475-2891-9-36

**Published:** 2010-09-14

**Authors:** Marjolein Haftenberger, Thorsten Heuer, Christin Heidemann, Friederike Kube, Carolin Krems, Gert BM Mensink

**Affiliations:** 1 Robert Koch-Institute, Department of Epidemiology and Health Reporting, Postbox 65 02 61, D-13302 Berlin, Germany; 2 Max Rubner-Institute, Department of Nutritional Behaviour, Haid-und-Neu-Straße 9, D-76131 Karlsruhe, Germany

## Abstract

**Background:**

Validation of a food frequency questionnaire (FFQ) is important as incorrect information may lead to biased associations. Therefore the relative validity of an FFQ developed for use in the German Health Examination Survey for Adults 2008-2011 (DEGS) was examined.

**Methods:**

Cross-sectional comparisons of food consumption data from the FFQ and from two 24-hour recalls were made in a sample of 161 participants (aged 18 to 80 years) of an ongoing nationwide survey, the German National Nutrition Monitoring (NEMONIT). The data collection took place from November 2008 to April 2009.

**Results:**

Spearman rank correlations between the FFQ and the 24-hour dietary recalls ranged from 0.15 for pizza to 0.80 for tea, with two third of the correlation coefficients exceeding 0.30. All correlation coefficients were statistically significant except those for pizza and cooked vegetables. The proportion of participants classified into the same or adjacent quartile of intake assessed by both methods varied between 68% for cooked vegetables and 94% for coffee. There were no statistically significant differences in food consumption estimates between both methods for 38% of the food groups. For the other food groups, the estimates of food consumption by the FFQ were not generally higher or lower than estimates from the 24-hour dietary recalls.

**Conclusions:**

The FFQ appears to be reasonably valid in the assessment of food consumption of German adults. For some food groups, such as raw and cooked vegetables, relative risks estimates should be interpreted with caution because of the poor ranking agreement.

## Background

Many epidemiological studies investigate the effects of diet on health in large populations. For such studies, accurate methods to assess middle or long-term dietary intake are needed. However, comprehensive dietary methods are often expensive, time consuming and request a high commitment of participants [1]. Self-administered food frequency questionnaires (FFQ) ask respondents about the frequency and often about the portion size of a limited number of usually consumed foods. Within large health surveys, which primarily give a broad representative overview of the actual health situation within a specified population, the dietary assessment methods should be feasible to apply next to assessments of other health relevant topics. FFQs measure usual intake over a middle or long-term period, which is highly relevant for the survey objective of monitoring usual behaviour. In comparison to other dietary intake assessment methods, FFQs are relatively inexpensive, easy and quick to administrate [2,3]. Their ability to compare groups or rank persons according to their intake of major food groups is often sufficient for health survey purposes and therefore FFQs are often the method of choice for such surveys [3]. However, only a limited number of foods can be included in an FFQ, for feasibility reasons and to limit the burden for participants. This predefined food list may have to be adapted to the population of interest and to actual food habits [3]. Like all dietary assessment methods, FFQs are prone to measurement errors and it is highly recommended to validate new FFQs [1-3].

A self-administered, semi-quantitative FFQ was developed to assess usual food consumption within the German Health Examination Survey for Adults 2008-2011 (DEGS) [4]. The relative validity of this questionnaire was studied among participants of another nationwide survey, the German National Nutrition Monitoring (NEMONIT). This sample was chosen, because they were already recruited and interviewed by trained interviewers using the 24 h-recall method [5].

## Methods

The German Health Examination Survey for Adults 2008-2011 (DEGS) aims to monitor overall health status and its determinants in the German population aged 18 to 79 years in a cross-sectional component and aged 18 years and over in a longitudinal component [4]. The FFQ is sent to the subjects by post prior to a local examination visit.

To assess the relative validity of the DEGS questionnaire, food consumption derived from the FFQ was compared with intakes assessed by two 24-hour dietary recalls among participants of the German National Nutrition Monitoring (NEMONIT). This is a longitudinal survey in a sample of about 2000 participants recruited from the German National Nutrition Survey II. NEMONIT aims to monitor food consumption in the German adult population aged 18 to 80 years, which is the same population the FFQ was developed for. From the participants, each year two 24-hour dietary recalls are administered on randomly drawn days within a period of three months, in four consecutive waves [5]. Between the end of November 2008 and February 2009, 487 participants completed both 24-hour recalls and 209 of them completed at least one of those recalls between the 9th of January and the 16th of February 2009. To ensure that both methods approximately reflect the same reference period of four weeks, the FFQ was sent to those 209 participants at the end of February immediately after completion of both recalls. This group included men and women aged 18 to 79 years. The participants were asked to return the completed questionnaire preferably within the next week. To improve the response, they received a gift voucher of value 7.50 € upon completion of the FFQ. In total, 164 participants returned the completed FFQ. Three participants were excluded from the analysis, because missing values for food items exceeded 25%. In total, 161 participants (77%) were included in the data analysis. For 58 participants (36%), at least one of the days of the recalls was a special day of food consumption due to feasts, travels, holidays, illness or shift work. The time between the completion of the FFQ and the 24-hour recalls ranged from 24 to 125 days (mean 55 days) for the first recall and from 10 to 89 days (mean 35 days) for the second recall. For five participants, both recalls were within the reference period of the FFQ (28 days). Furthermore, 55 participants completed their second recall within that reference period.

The surveys were approved by the German federal data protection office. Respondents were informed in detail about the study objectives, interview and examination procedures as well as the handling of data records and analyses under pseudonymous conditions. It was made clear that participation was on a voluntary base and could be terminated at any time. For the NEMONIT and for the validation study all participants provided written informed consent.

### The Food Frequency Questionnaire

The FFQ is a revision of the questionnaire used in the German Health Interview and Examination Survey for Children and Adolescents (KiGGS). The latter questionnaire was developed with consultation of experts involved in large previous surveys about the food list and designed using elements from and considering cognitive criteria of the National Cancer Institute Food Frequency Questionnaire [6]. Design elements include the application of consecutive questions instead of a grid format, the use of pictures to illustrate standard portions, standard use of 10 frequency categories (11 in DEGS), varying answer categories for portion size and using additional questions on specific foods (like fat content of milk). The questionnaire for children is described in detail elsewhere [7] and showed a good compliance in KiGGS [8]. For DEGS, the children's questionnaire was primarily revised to adapt the food item list for use in the adult population. To select the food items, dietary intake data from previous surveys (the German National Health and Examination Survey 1998 [9] and the German National Nutrition Survey II [10] performed between 2005 and 2007) were analysed to detect the most frequently consumed foods. Finally, the FFQ was cross-checked by several nutrition and public health experts for completeness of relevant foods and usability. The FFQ was pre-tested and showed a good usability. The feedback experience from the pretest and the main study DEGS was positive and participants had no problems to classify their consumed foods.

The FFQ includes questions about the frequency and the amount of 53 food items, consumed during the past four weeks. The questionnaire was sent to the participants by mail with the request to complete the questionnaire at home and to return it. Frequency of consumption of food items was asked according to specified categories. The frequency categories were: never, once a month, two to three times a month, one to two times a week, three to four times a week, five to six times a week, one time per day, two times per day, three times per day, four to five times per day and more than five times per day. In addition, the respondents had to indicate the portion sizes of the food items consumed in predefined answering categories. Pictures were used to aid the estimation of portion size for 33 food items. Some questions about vegetarian and cooking habits were included but not analysed here. Supplement use was assessed in a computer aided interview within DEGS and was therefore not part of the FFQ. For the analysis presented here, similar food items were categorised in one group. For example the food items 'honey and marmalade' and 'chocolate spread' were grouped as 'sweet spreads', or the food items 'fried and curried sausages' and 'doner kebab and hamburger' as 'fast food'. In total, 29 food groups will be presented.

### 24-Hour Dietary Recalls

For each 24-hour dietary recall, participants were asked by telephone on randomly drawn days about their food consumption during the previous day in detail. The two interviews of each participant were at least one week but no more than three months apart. The 24-hour dietary recalls were equally distributed over all days of the week and the weekend. Recalls for Saturdays were performed on the following Monday. Trained interviewers of a marketing research institute performed the 24-hour dietary recalls. For the interviews, the software EPIC-SOFT was used. EPIC-SOFT was developed as a calibration instrument for the European Prospective Investigation into Cancer and Nutrition Study by the International Agency for Research on Cancer (IARC) [11,12]. The version applied was adapted to the German dietary habits and modified for the use in the German National Nutrition Survey II[13] and NEMONIT [5]. In EPIC-SOFT, personal characteristics, such as self-reports of weight and height, and information on a special diet (e.g. vegetarian) or a special day (e.g. travel), were registered. At the beginning of the interview, food items consumed by the participant during the previous day were briefly listed using a quick list. Thereafter, each food item was specified in detail (for example preparation methods, recipes and brand names). To assist participants to indicate the consumed amount of a food, a picture booklet providing different photographed portion sizes for various foods and dishes was used [11,13]. This was a short adapted version of the original EPIC-SOFT picture book. A trained assistant revised incomplete data from the recalls. Intake of each food item was calculated as the average intake of both recall days. The recalled food items were assigned to the food groups fitting with the food groups defined by the FFQ.

### Data and Statistical Analysis

Twenty-three participants had some missing values (maximal four) for frequency of intake on food items in the FFQ. These participants were excluded from the analysis of that particular food item. This explains the differences in number of participants for particular analyses. For participants with reported frequency, missing values for portion size were replaced by the mean portion size of the other participants for that food item (n = 8). If frequency of consumption of a food item was reported as never, but portion size was given, the intake of this food item was assumed to be zero. Participants, who reported both a high frequency of consumption (≥ four or five times a day) and a high portion size (four or more glasses/cups) for water, tea and non-alcoholic beverages on the FFQ, resulting in implausible high amounts, were excluded from the analysis of the particular item.

For most food groups, the food consumption was not normally distributed. Therefore, non-parametric methods were used to evaluate the validity of the FFQ relative to the 24-hour recalls. Spearman rank order correlation coefficients [14] were calculated for all participants and stratified by sex and age group. In a separate analysis, the coefficients were calculated excluding participants with special days of consumption in their recalls. In addition, participants were grouped into quartiles for each food group, to test the agreement in ranking participants regarding their food consumption as estimated from both methods. The proportion of participants classified into the same, adjacent or opposite quartile for both methods was calculated. The degree of agreement was evaluated by the weighted kappa coefficient [14]. This analysis was not applied for food groups where more than 25% of the participants had a zero consumption of these foods in their recalls which disabled the construction of quartiles. Mean intakes derived from both methods and differences of intakes of food groups between both methods are presented. The significance of differences in intake of food groups between the FFQ and the average of both 24-hour recalls was tested with the Wilcoxon sign rank test [14]. All analyses were performed with SAS Version 9.2 (SAS Institute Inc., Cary, NC).

## Results

### Sample Characteristics

Characteristics of the validation sample are shown in table 1. Fifty-one percent of the participants were men. Mean age of the participants was 51 years. The majority of the participants was 35 to 64 years old (70%). Only 14% of the participants were younger than 35 years old and 16% were 65 years and older. Mean body mass index (BMI), based on self-reports of weight and height, was 26.1 kg/m^2 ^in men and 25.3 kg/m^2 ^in women. Sixty-two percent of the men and 43% of the women were overweight or obese (BMI ≥ 25.0 kg/m^2^).

**Table 1:** Distribution of age and body mass index groups in the validation sample (n = 161)

	Men (n = 82)		Women (n = 79)	
	
	n	%	n	%
Age group				
18 - 34 years	11	13.4	11	13.9
35 - 50 years	28	34.2	29	37.2
51 - 64 years	29	35.4	27	34.6
65 - 79 years	14	17.1	12	15.4
				
BMI^a^				
Under-/normal weight (BMI < 25.0 kg/m^2^)	31	37.8	45	57.0
Overweight (BMI: 25.0 - 29.9 kg/m^2^)	42	51.2	23	29.1
Obesity (BMI ≥ 30.0 kg/m^2^)	9	11.0	11	13.9

a According to WHO classification [23]

### Correlations

The correlations of the estimates of food consumption between both methods were moderate to high for most food groups (Table 2). High correlation coefficients (≥ 0.70) were observed for tea, coffee and butter/margarine. The correlation coefficients were moderate (0.40 to 0.69) for sweet spreads, milk, breakfast cereals, alcoholic and non-alcoholic beverages, meat products, fresh fruits, water, bread, sweets, cream cheese, cheese and curd cheese/soured milk/yoghurt. Only for the food groups cooked vegetables and pizza, the correlation was not significant.

**Table 2:** Spearman rank correlation coefficients of food group intake between the food frequency questionnaire and two 24-hour dietary recalls

		Sex		Age group			
		
	All participants	Men	Women	18- 34 years	35 - 50 years	51 - 64 years	65 - 80 years
Food group	n = 161	n = 82	n = 79	n = 22	n = 57	n = 56	n = 26
Tea	0.80‡	0.74‡	0.76‡	0.74‡	0.79‡	0.81‡	0.83‡
Coffee	0.78‡	0.69‡	0.83‡	0.75‡	0.81‡	0.71‡	0.76‡
Butter and margarine	0.70‡	0.67‡	0.67‡	0.80‡	0.61‡	0.66‡	0.71‡
Sweet spreads	0.65‡	0.66‡	0.63‡	0.26	0.62‡	0.52‡	0.69‡
Milk	0.63‡	0.61‡	0.64‡	-0.03§	0.67‡	0.70‡	0.72‡
Breakfast cereals	0.62‡	0.64‡	0.60‡	0.58†	0.57‡	0.63‡	0.75‡
Non alcoholic beverages	0.61‡	0.62‡	0.59‡	0.70‡	0.60‡	0.64‡	0.29
Alcoholic beverages	0.60‡	0.65‡	0.53‡	-0.07§	0.52‡	0.73‡	0.87‡
Meat products	0.58‡	0.45‡	0.49‡	0.62†	0.58‡	0.51‡	0.55†
Water	0.53‡	0.54‡	0.52‡	0.38	0.44‡	0.52‡	0.66‡
Fresh fruits	0.51‡	0.55‡	0.46‡	0.51*	0.54‡	0.53‡	0.38
Curd cheese, soured milk, yoghurt	0.49‡	0.41‡	0.48‡	0.24	0.49‡	0.47‡	0.40*
Bread	0.46‡	0.41‡	0.40‡	0.31	0.46‡	0.43‡	0.56*
Sweets	0.45‡	0.45‡	0.45‡	0.30	0.32*	0.53‡	0.63‡
Cream cheese	0.40‡	0.32†	0.48‡	0.41	0.46‡	0.34†	0.37
Cheese	0.40‡	0.40‡	0.39‡	0.38	0.52‡	0.25	0.30
Fish	0.36‡	0.38‡	0.32†	0.22	0.42‡	0.38†	0.22
Salty snacks	0.33‡	0.33†	0.33†	0.62†	0.17	0.34†	0.33
Meat	0.29‡	0.32†	0.21	0.13	0.13	0.28*	0.62‡
Raw vegetables	0.29‡	0.23*	0.27*	0.18	0.43†	0.24	0.18
Processed fruits	0.27‡	0.16	0.38‡	0.17	0.18	0.35†	0.41*
Potatoes	0.27‡	0.26*	0.26*	0.36	0.17	0.12	0.47*
Fast food	0.25‡	0.16	0.36*	0.43*	0.40*	0.11	0.07
Legumes	0.22‡	0.28*	0.09	0.26	0.22	0.22	0.19
Eggs	0.20†	0.28*	0.10	-0.23§	0.33*	0.15	0.44*
Pasta	0.19*	0.27*	0.09	0.16	0.20	0.14	0.26
Rice	0.19*	0.18	0.20	-0.15§	0.15	0.35†	0.11
Cooked vegetables	0.16	0.08	0.23*	-0.16§	0.26	0.21	0.00§
Pizza	0.15	-0.10§	0.19	0.10	-0.05§	0.07	-§

Significance level: * *P *< 0.05, †*P *< 0.01, ‡ *P *< 0.001

§ The correlation coefficient was negative or could not be calculated due to a high number of participants who did not consume the food on both recall days

After exclusion of participants, who recalled their food consumption of at least one day of special consumption, the spearman correlation coefficients were similar or higher for most food groups. The correlation coefficient for cooked vegetables improved remarkably and became significant. However, the correlation coefficient for breakfast cereals, pizza and pasta decreased with the correlation for pasta being no longer significant (data not shown).

For men and women, the correlation coefficients were similar. However, for some food groups, there were discrepancies between men and women. This especially concerned those food groups with lower correlation coefficients. The correlation coefficients for meat, legumes, eggs and pasta were only significant in men. Whereas, the correlation coefficients for processed fruits, fast food and cooked vegetables were only significant in women.

For most food groups, the age groups 35-50, 51-64 and ≥ 65 years showed similar correlations. Among the participants younger than 35 years, the correlations were lower for several food groups as compared to the higher age groups, especially for sweet spreads, milk, alcoholic beverages, water, curd cheese/soured milk/yoghurt, bread, sweets and meat, but stronger for snacks and fast food. Interestingly, the spearman correlation coefficients for alcoholic beverages and sweets increased with age.

### Ranking Misclassification

The degree of potential misclassification associated with categorised intakes assessed by the FFQ in comparison to the 24-hour dietary recalls was examined as the proportion of participants classified in the same, adjacent, or opposite quartile (table 3). For tea, breakfast cereals, alcoholic beverages, cream cheese, cheese and curd cheese/soured milk/yoghurt, eggs, fast food, fish, salty snacks, pasta, rice, processed fruits, legumes and pizza ranking into quartiles was not possible, since more than 25% of the subjects did not consume these foods on each recall day. For the other 14 groups, the proportion of participants classified within the same or the adjacent quartile ranged from 68% for cooked vegetables to 94% for coffee. Classification into the opposite quartile was 10% or less for all food groups, with highest levels of opposite classification for meat (10%), raw vegetables (10%), potatoes (9%) and cooked vegetables (7%). Except for cooked vegetables, the weighted kappa coefficients were significant for all food groups. Exclusion of participants with special days of consumption in their recalls did not improve the agreement for cooked vegetables (data not shown). A moderate to good agreement in ranking the participants according to their intake between methods (weighted kappa > 0.40) was observed for coffee, butter and margarine, sweet spreads, non-alcoholic beverages and milk. An acceptable agreement (kappa 0.20 to 0.39) was seen for meat products, water, fresh fruits, bread and sweets.

**Table 3:** Agreement of quartiles for food group intake assessed by the food frequency questionnaire and two 24-hour dietary recalls*

	Agreement of quartiles for food group intake (all participants)				
	
Food group	Same quartile%	Adjacent quartile%	Opposite quartile%	Weighted Kappa	95%- Confidence Interval
Coffee	50.3	44.1	0.0	0.54	0.45 - 0.62
Butter and margarine	50.3	40.4	0.6	0.51	0.42 - 0.60
Sweet spreads	48.4	39.0	1.3	0.49	0.40 - 0.59
Milk	45.6	40.3	1.9	0.45	0.35 - 0.55
Non alcoholic beverages	49.0	33.6	3.4	0.45	0.34 - 0.56
Meat products	40.4	44.7	1.9	0.39	0.29 - 0.49
Water	38.4	44.2	2.2	0.36	0.25 - 0.47
Fresh fruits	39.7	37.9	4.9	0.34	0.23 - 0.44
Sweets	40.2	39.0	5.0	0.31	0.20 - 0.43
Bread	38.6	39.9	5.2	0.29	0.18 - 0.41
Raw vegetables	29.8	45.0	10.1	0.18	0.07 - 0.29
Meat	29.4	40.6	10.0	0.15	0.04 - 0.26
Potatoes	30.6	38.2	9.4	0.15	0.04 - 0.26
Cooked vegetables	31.0	37.3	7.0	0.09	-0.03 - 0.20

*Analysis was not performed for food groups with more than 25% of the participants who did not consume this food in the 24-hour dietary recalls.

### Comparison of mean intakes

Table 4 shows the mean food group intakes estimated by both methods. The mean intake of legumes, rice, potatoes, raw vegetables, cheese, curd cheese/soured milk/yoghurt, fresh fruits, non-alcoholic beverages and milk estimated from the FFQ was significantly higher than the intake assessed by the 24-hour recalls. The intake of coffee, meat products, sweets, butter and margarine, sweet spreads, fish, processed fruits, cream cheese and pizza obtained by the FFQ were lower as compared to the 24-hour dietary recalls. Food consumption estimated by the FFQ was not generally higher or lower than estimates from the 24-hour dietary recalls. The mean intake of eleven food groups did not show significant differences between both methods.

**Table 4:** Food intake assessed by the food frequency questionnaire and two 24-hour dietary recalls and differences between both methods: mean and standard deviation (SD)

	Food intake (g/d)							
		
		Food frequency questionnaire		24-hour dietary recalls		Differences FFQ - 24-hour dietary recalls		
		
Food group	N	Mean	SD	Mean	SD	Mean	SD	P*
Coffee	159	403.5	343.2	509.4	406.3	-105.8	313.3	< 0.001
Alcoholic beverages	155	126.8	183.8	174.6	277.4	-47.8	180.6	0.059
Tea	159	324.6	479.2	350.9	493.4	-26.3	374.2	0.161
Meat products	161	29.7	28.0	44.3	45.7	-14.6	43.3	< 0.001
Sweets	159	50.9	52.0	65.4	62.9	-14.5	63.2	0.008
Pasta, noodles	161	23.8	21.6	34.1	61.8	-10.3	58.2	0.896
Butter and margarine	161	8.3	9.4	17.7	17.8	-9.4	14.7	< 0.001
Sweet spreads	159	9.8	10.2	18.8	22.4	-8.9	18.7	< 0.001
Fast food	159	10.5	15.7	18.3	41.2	-7.9	38.1	0.626
Fish	161	16.9	19.0	21.5	49.9	-4.7	49.2	0.026
Breakfast cereals	161	3.6	7.2	7.2	19.9	-3.6	16.6	0.857
Processed fruits	160	6.6	11.9	10.1	32.0	-3.5	31.0	0.029
Pizza	160	14.6	19.4	16.9	62.5	-2.2	63.1	< 0.001
Salty snacks	161	4.6	9.0	6.2	15.4	-1.6	14.0	0.099
Bread	153	143.6	98.5	144.7	76.6	-1.1	94.8	0.401
Eggs	161	12.5	10.9	13.5	23.3	-0.9	23.5	0.419
Cream cheese	161	4.3	8.0	4.7	14.8	-0.4	13.0	0.002
Legumes	158	14.3	21.9	13.8	36.9	0.5	40.5	0.003
Meat	160	52.7	46.4	52.1	58.6	0.6	66.0	0.501
Cooked vegetables	158	54.6	51.5	50.9	51.7	3.7	68.7	0.474
Cheese	161	26.2	25.9	16.2	18.2	9.8	25.5	< 0.001
Rice	161	19.2	21.6	9.1	27.4	10.1	30.3	< 0.001
Potatoes	160	85.8	57.1	70.6	82.7	15.2	81.8	< 0.001
Raw vegetables	158	72.9	81.8	39.4	48.8	33.5	77.3	< 0.001
Curd cheese, soured milk, yoghurt	160	95.1	107.3	57.6	86.7	37.5	111.4	< 0.001
Non alcoholic beverages	149	357.8	473.0	288.2	378.4	69.6	394.3	0.003
Fresh fruits	161	247.7	81.8	174.9	174.2	72.8	280.0	< 0.001
Water	138	842.3	608.7	743.3	514.1	99.0	594.6	0.292
Milk	158	220.2	244.7	111.5	170.3	108.7	249.6	< 0.001

* Sign rank test

## Discussion

The DEGS-FFQ showed a reasonable to good agreement in ranking of participants towards their intake for most food groups compared to two 24-hour dietary recalls. The spearman's correlation coefficients for intakes measured by the FFQ and the 24-hour dietary recalls ranged between 0.15 and 0.80 with most values of 0.30 and higher. The observed correlation coefficients were in a similar range as observed in other validation studies [15-20]. For example, a validation of the FFQ used in the German EPIC cohort showed Spearman rank correlation coefficients between the FFQ and twelve 24-hour dietary recalls between 0.14 and 0.90 [17]. Although a direct comparison is difficult because of differences in food classification, similar correlations were seen for coffee and tea, bread, salty snacks, spreads, fruits, legumes, potatoes, soft drinks, milk, cheese and processed meat. In our study higher correlations were seen for cereals, desserts and fish and lower for vegetables, eggs, sweets and biscuits, alcoholic beverages and meat.

Despite a reasonable agreement for ranking the participants according to their intake based on both methods, we observed considerable differences in absolute intakes between the FFQ and the 24-hour dietary recalls for particular food groups. However, there is no evidence that the FFQ systematically over- or underestimates food (group) intake in comparison to the 24-hour recall method.

Some disagreement of food intake estimates was expected because of the different reference time of both methods. Discrepancies were especially expected for foods consumed rarely, because the probability of assessing such foods on the two recall days is low. More recalls per person would probably lead to a higher agreement with the FFQ [1].

A multiple day weighed record was also considered as the comparative method since it has few correlated errors with the FFQ [3], but it also would have a divergent reference period and is logistically less feasible than the 24-hour recall. The dietary history method has similar sources of error as an FFQ and is therefore inappropriate. Biomarkers were not considered because they are nutrient specific [3] and this FFQ was primarily designed to measure food intake. The FFQ and the 24-hour dietary recall have some similar error sources, like the reliance on memory and the perception of portion sizes [1,3]. However, the FFQ stresses long-term memory and the 24-hour recall short-term memory. In addition, the 24-hour recall method was interviewer-based using open-ended questions, whereas the FFQ was self-administered with close-ended questions. Such differences let us assume that the errors are sufficiently independent and that the 24-hour recall method is an adequate comparison method.

The validation study was performed in the ongoing survey NEMONIT which offered many advantages. Participants were allready available and motivated to participate. Accordingly, the response rate was high (77%). The validation study could be realized in a short time and resources could be saved. Nevertheless, there are some limitations of this design.

A main limitation was the tight time schedule of the validation study. Ideally, the 24-hour recalls should be completed within the reference period of the FFQ [3]. In our validation study, only a few participants completed both recalls within the reference period. In an additional analysis, we compared spearman correlation coefficients and mean differences in four intervals of time between completion of the 24-hour recall and the FFQ (within 28 days, 29 to 42 days, 43 to 56 days, more than 56 days), which showed only small differences in relative validity. An exception was the intake of alcoholic beverages for which the underestimation by the FFQ compared to the 24-hour recalls increased with increasing time lag (data not shown). An explanation may be that participants with a longer time interval between both methods more often had a recall day in the Christmas period, in which drinking habits may differ from usual intake. It was shown previously that FFQs may give reasonably good estimates of average amounts of usual alcohol intake, but they are not sensitive for day to day variability in drinking habits [21]. However, infrequent drinkers may report either no drinks within the 24-hour recalls or, as may be the case in our study, report unusual high intake.

A consequence of performing the validation study in the ongoing survey seems to be some degree of selection of the participants. All ages, for which the FFQ was developed, were represented. However, the majority of the participants were between 35 to 64 years old and the proportions of participants younger than 35 years or older than 64 years were rather small. The age-stratified analysis showed that the correlation coefficients for several food groups were somewhat weaker and less often significant among the participants younger than 35 years compared to the other age groups. On the other hand, for some foods, like fast food, salty snacks and non-alcoholic beverages, the correlations were highest in the youngest age group. This is probably because, fast food and non-alcoholic beverages are consumed more often on a regular base among younger compared to older people.

The weak associations for some food groups may in part be due to the within-subject variance in the 24-hour dietary recalls. The calculation of deattenuated correlation coefficients to correct for intra-individual variability is used in many studies [15,17]. Accordingly, deattenuated spearman correlation coefficients are generally higher than the attenuated correlation coefficients. However, due to the feasibility of only two recalls and the non-normal distribution of the intake data (even after log-transformation) in our study we did not present deattenuated correlation coefficients.

The weak relative validity for raw and cooked vegetables should be discussed, since many studies focus on the effects of vegetable consumption on various outcomes. The food group of vegetables includes a considerable number of various products, consumed either as single foods or as part of mixed dishes. In the FFQ, vegetable consumption is assessed by two global questions. It is indicated, that global questions on vegetable consumption rather underestimate the vegetable consumption, whereas a higher number of questions on differently prepared vegetables rather result in agreeable levels of intake compared to six day dietary records and four 24-hour dietary recalls [20]. For future development of FFQs, it should be considered to inquire vegetable consumption by multiple detailed questions to increase the level of precision. However, it was previously noted that there is a decreasing marginal gain in information when elongating the number of food items in an FFQ after a certain point [3]. The DEGS-FFQ is relatively short since it should not discourage to participate in the main DEGS examination.

There are some possible explanations for the considerable differences of average food intake estimates of some food groups between both methods. One is the estimation of portion sizes. The FFQ used close-ended questions with predefined portion sizes, while in the recalls the consumed amounts are quantified as detailed as possible. In addition, the broad food groups in the FFQ may complicate it sometimes to average the portion size. For instance, lettuce and cucumber both belong to the group of raw vegetables, but have very different specific weights. Following, the application of a predefined average weight for such food group portions might result in measurement errors. Besides, many foods, such as vegetables, meat and potatoes, are often consumed as part of mixed dishes. The FFQ inquired the frequency and amount consumed of single food items. Therefore, the FFQ relies on the participant's ability to quantify the consumption of a food from single foods as well as from mixed dishes. In contrast, the foods consumed as part of mixed dishes were quantified separately in the recalls. For milk, the relatively large difference in means (despite an acceptable agreement for ranking) may be caused by the use of different standard portion sizes. The question on milk in the FFQ includes both milk as a plain beverage and milk added to coffee and breakfast cereals. This may have complicated the estimation with predefined portion sizes and may partly explain the observed difference. In future, a separate question for milk in coffee and milk as a beverage would make sense. The differences in the applied standard portion sizes are probably a source of different findings and harmonizing portion sizes is an important subject for future work. The FFQ could be used within NEMONIT for assessing infrequent and non-consumers of specific foods to improve the estimation of usual intake distribution from the recalls [22]. Within DEGS the results will be useful to improve the evaluation of risk analyses, for instance through sequentially calibrating the food frequency intakes with estimates of the recalls.

The effect of diet on a health outcome is most frequently quantified as odds ratio or relative risk in epidemiological studies. Therefore, FFQs must be able to rank individuals along the distribution of intake to provide accurate risk estimates. The validation study of the DEGS-FFQ showed a moderate to good agreement for ranking participants towards their intake for the food groups tea, coffee, butter and margarine, sweet spreads, milk, breakfast cereals, alcoholic and non-alcoholic beverages, meat products, fresh fruits and water. In addition, rankings were reasonable for dairy products, bread and sweets.

## Conclusions

The DEGS-FFQ seems to be a valid instrument for both genders and the investigated age groups. It may be reasonably well used to assess the relative risk of food consumption quantiles for most food groups. For some food groups, such as raw and cooked vegetables, relative risks estimates should be interpreted with caution because of the poor ranking agreement. However, we observed considerable differences in absolute intakes estimated by both methods with no evidence that either the FFQ or the 24-hour recalls better reflect real food consumption. Therefore, the use of absolute intake estimates from the FFQ should also be considered with care and the sensitivity to standard portion size use should be further investigated. In addition, food consumption information from the FFQ is suitable to be used as confounding factors in exposure-outcome analyses.

## Competing interests

The authors declare that they have no competing interests.

## Authors' contributions

GM and CH designed the study and wrote the study proposal. MH and TH coordinated the field work. TH, CK, MH and FK collected and managed the data. MH and FK performed the statistical analysis. MH wrote the manuscript. All authors critically revised the manuscript, read and approved the final manuscript.
